# Acute Esophageal Necrosis in a Patient With Severe Cardiovascular Disease and Arrhythmia

**DOI:** 10.7759/cureus.65691

**Published:** 2024-07-29

**Authors:** Sonia Samuel, Anthony Passarella, Rashelle Tsyrlin, Stephen Hasak, Grigoriy Gurvits

**Affiliations:** 1 Internal Medicine, Albany Medical Center, Albany, USA; 2 Gastroenterology and Hepatology, Albany Medical Center, Albany, USA; 3 School of Medicine, St. George's University School of Medicine, True Blue, GRD; 4 Gastroenterology and Hepatology, New York University (NYU) Langone Health, New York, USA

**Keywords:** atrial fibrillation, upper gastrointestinal bleed, gurvits syndrome, black esophagus, acute esophageal necrosis

## Abstract

Acute esophageal necrosis is a gastrointestinal syndrome characterized by diffuse, circumferential, black-appearing mucosa of the distal esophagus and involves various lengths. It is a multifactorial condition involving hypoperfusion from a low flow state, large reflux of gastric contents, and a poor mucosal barrier. Complications involve esophageal stenosis or stricture and esophageal perforation. Treatment is often supportive with correction of underlying conditions, aggressive fluid resuscitation, antacid therapy, and restriction of oral intake. We present an unusual case of black esophagus in a patient with significant cardiovascular disease and rhabdomyolysis and discuss its pathogenesis, management, and outcome.

## Introduction

This article abstract was previously presented as a poster at the 2023 American College of Gastroenterology Annual Scientific Meeting on October 23, 2023.

Black esophagus (BE), acute esophageal necrosis (AEN), or Gurvits syndrome (GS) is a gastrointestinal condition characterized by the striking endoscopic finding of diffuse, circumferential, black-appearing mucosa extending from the gastroesophageal junction (GEJ) to involve various lengths. Rapid advances in endoluminal evaluation in the 21^st^ century have led to its increased recognition as an important cause of upper gastrointestinal bleeding. Its etiology is multifactorial: a combination of ischemic tissue hypoperfusion from a low flow state, massive influx of corrosive gastric contents, and decreased efficacy of the esophageal mucosal barrier. Risk factors include advanced age, cardiovascular disease, malignancy, diabetic ketoacidosis, thromboembolic phenomena, and malnourishment. Endoscopy is diagnostic. Complications are uncommon and may include late-onset esophageal stenosis or stricture in up to 10% of cases. BE carries a poor prognosis with 32% mortality from comorbid conditions [1,2]. We present a novel case of BE in an elderly patient with significant cardiovascular disease and rhabdomyolysis and discuss its pathogenesis, management, and prognosis. 

## Case presentation

**Figure 1:**
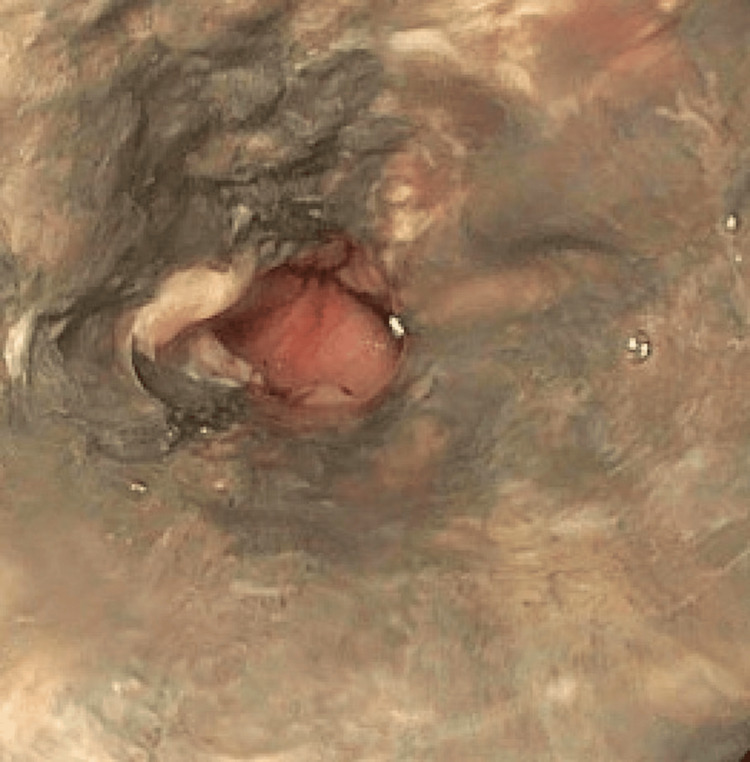
Necrosis of the distal esophagus

A 68-year-old male presented to the hospital with worsening bilateral lower extremity pain and numbness. His medical history was notable for atrial fibrillation without anticoagulation and active tobacco use. His blood pressure was 97/60 mmHg, heart rate 79 beats per minute, and his abdomen was non-tender on physical examination. Laboratory analysis revealed white blood cell count of 18,000/uL, hemoglobin of 16.9 g/dL, platelets of 98,000 uL, blood urea nitrogen of 16 mg/dL, serum creatinine of 0.83 mg/dL, albumin of 3.0 g/dL, alanine aminotransferase of 252 IU/L, aspartate aminotransferase of 689 IU/L, and serum creatinine kinase of 30646 IU/mL. He was found to have extensive multivessel occlusive disease, was placed on a heparin drip, given aggressive fluid resuscitation, and admitted to the intensive care unit. The patient underwent an emergent axillary bifemoral bypass with bilateral lower extremity four-compartment fasciotomies followed by left lower extremity below-the-knee amputation the following day. His postoperative course was complicated by atrial fibrillation with rapid ventricular response requiring beta blockade. On postoperative day 4, he was noted to have pink watery bowel movements with a decrease in his hemoglobin to 8.6 g/dL. The colonoscopy was unremarkable. His nasogastric tube (NGT) returned coffee grounds fluid and hemoglobin decreased further to 6.6 g/dL. Emergent esophagogastroduodenoscopy (EGD) revealed circumferential discoloration of the esophageal mucosa with confluent ulceration starting from the GEJ and extending proximally to 20 cm from central incisors (Figure 1).

The patient was transfused two units of packed red blood cells, started on intravenous twice daily pantoprazole, kept nil per os, and heparin drip was discontinued. Over the course of the next two days, his hemoglobin remained stable. So the patient was started on a clear liquid diet and eventually transitioned back to a heparin drip. His hospital course was further complicated by hemoptysis, endotracheal intubation for respiratory failure, and multiple unsuccessful attempts at NGT tube placements. Repeat endoscopy on postoperative day 18 demonstrated resolution of the black esophagus and a large blood clot attributed to NGT trauma. The patient was eventually discharged to rehabilitation on a regular diet and maintenance anticoagulation with apixaban after a two-month hospital course.

## Discussion

BE, AEN, or GS is an important gastrointestinal syndrome with increased recognition as the fourth leading cause of upper gastrointestinal bleeding in hospitalized patients [3]. Clinically, GS develops in the setting of hypoperfusion from hemodynamic instability (seen in cardiac arrhythmias, septic shock, hypovolemia), overflow of corrosive gastric contents (seen in alcohol intoxication, diabetic ketoacidosis, duodenal ulcer disease), and compromise of local esophageal mucosal defenses (seen in a malnourished state, immunodeficiency, malignancies). Risk factors include age, male gender, cardiovascular and renal disease, alcohol abuse, diabetes mellitus, hypercoagulable state, general debilitation, hiatal hernia, aortic aneurysm, and trauma. A paucity of blood supply in the distal esophagus predisposes it to ischemic insult and accounts for almost universal (97%) predilection in presentation. Around 90% of patients with BE present with upper gastrointestinal hemorrhage (hematemesis, coffee-ground emesis, and melena). Others may experience nausea, vomiting, abdominal pain, and dysphagia but asymptomatic cases have also been described in the literature [4]. Endoscopic appearance is classic: a striking diffuse, circumferential, black-appearing mucosa extending from the GEJ and involving various lengths. Associated findings may include duodenal ulcer disease and gastric outlet obstruction in up to 25% of cases [1]. Tissue biopsy is not required but may be helpful in excluding fungal or viral infections in immunocompromised individuals [4,5]. Staging of GS aids in histopathologic understanding of the disease and prognostic expectations as well as endoscopic surveillance strategies and management of late complications. Treatment of BE is largely supportive and aimed at correcting underlying comorbid conditions, hemodynamic support with volume resuscitation, nil per os restriction, and high-dose intravenous proton pump acid inhibitor therapy. Broad-spectrum antimicrobial coverage and emergent surgical consultation should be sought in immunocompromised patients and in those with concern for esophageal perforation with acute clinical decompensation, which is seen in less than 7% of cases. Late complications include esophageal stricture or stenosis and may occur in nearly 10% of patients, requiring repeated endoscopic dilatations [4,6]. Overall, the prognosis of BE is poor as nearly one-third of patients succumb to the underlying medical conditions. However, mortality specific to BE is close to 6% and is highest in patients with diabetic ketoacidosis, esophageal perforation, and a compromised immune system. Repeat endoscopy should be considered in two months to document expected mucosal healing and rule out occult pathology that could have been obscured by necrosis on index EGD [7,8]. 

Our patient had significant cardiovascular disease with postoperative arrhythmia and underlying tobacco-related atherosclerosis that compounded the immediate bypass effect of axillary femoral revascularization, resulting in poor perfusion. The vascular compromise and malnourishment with decreased mucosal defenses led to GS. Conservative management with hemodynamic resuscitation, correction of comorbidities, and high-dose intravenous antiacid therapy resulted in the resolution of BE and allowed an opportunity to witness the natural course of this striking medical condition during the prolonged hospitalization.

## Conclusions

BE remains an intriguing condition with rising prevalence in hospitalized patients presenting with upper gastrointestinal hemorrhage. It is gaining widespread recognition both in medical wards and in gastrointestinal literature. Enhanced awareness of this disease process will enable clinicians to effectively manage and treat patients, thereby decreasing the rates of morbidity and mortality.
